# Area deprivation and multi-domain measures of ageing: biological ageing, cognitive, and subjective health across the entire adult age span

**DOI:** 10.1007/s10433-026-00937-6

**Published:** 2026-07-22

**Authors:** Wen Wang, Steven Haworth, Yanchun Bao, Meena Kumari

**Affiliations:** 1 Institute for Social and Economic Research, University of Essex, Colchester, UK; 2 Department of Internal Medicine, Division of Pulmonary, Critical Care and Sleep Medicine, University of Kansas Medical Center, Kansas City, KS USA; 3 School of Mathematics, Statistics and Actuarial Science, University of Essex, Colchester, UK

**Keywords:** Area deprivation, DNA methylation, Biological health score, Cognitive, Self-rated health

## Abstract

Area deprivation is a key contextual determinant of health, yet the consistency of effects on ageing processes over the adult life span remains insufficiently investigated. This study investigated associations between area deprivation and different ageing measures across the entire adult age span in Great Britain. We used data from the UK Household Longitudinal Study. A total of 13,174 participants in Health Assessment (2010–2012) and a DNA methylation subsample of 3,636 participants were included for cross-sectional analyses, as well as repeated measures of subjective functioning from subsequent 13-year follow-up for longitudinal analyses. Area deprivation was measured using the quintile of the Index of Multiple Deprivation, Townsend index, and Carstairs index. Ageing measures included DunedinPACE DNA methylation algorithm, Biological Health Score, cognitive function, and self-reported physical and general health. Linear regression models were used for cross-sectional analyses, and growth curve models were applied to examine longitudinal associations in subjective functioning. Subgroup analyses explored effect modification by individual-level sociodemographic characteristics. Higher area deprivation was associated with accelerated biological ageing, poorer cognitive performance, and worse subjective health, with clear and consistent social gradients across all deprivation measures. Longitudinally, individuals living in more deprived areas experienced persistently worse trajectories of physical and general health over time. Subgroup analyses suggest that area-level associations operate broadly across population groups, with few exceptions. These findings suggest that area-level socioeconomic disadvantage is a consistent contextual correlate of unequal ageing across adulthood that is associated with both baseline differences and, for some outcomes, divergent health trajectories over time.

**Supplementary Information:**

The online version contains supplementary material available at https://doi.org/10.1007/s10433-026-00937-6.

## Introduction

Ageing takes place within social and spatial contexts that shape opportunities, constraints, exposures, and resources across the life course. The characteristics of the neighbourhood in which people reside, including its population composition, built environment, and resources, are increasingly recognised as determinants of health and ageing. Framing the problem in terms of context and composition helps to clarify how neighbourhood effects might operate: Compositional explanations attribute differences to the characteristics of residents, whereas contextual explanations attribute differences to the features of places themselves, independent of who lives there (Macintyre et al. 2002; Cummins et al. 2007; Bambra 2022). While individual-level risk factors such as education, income, health behaviours, and genetic predisposition are well-established determinants of ageing outcomes, growing evidence indicates that where people live also matters. Contextual exposures may accumulate over time and produce biological, cognitive, and functional consequences that cannot be fully explained by individual-level characteristics (Stafford and Marmot 2003).

Area deprivation is a structural exposure that captures area-level inequality. In the UK, commonly used deprivation indices include the Index of Multiple Deprivation (IMD) (Ministry Of Housing, Communities & Local Government (2018 to 2021) 2015), the Townsend index (UK Data Service 2022), and the Carstairs index (Wheeler 2019; Brown et al. 2022), which differ in component variables and weighting but share the intent of quantifying relative advantage and disadvantage. Area deprivation is conceptualised as a contextual determinant because it captures material scarcity, service deficits, and concentrated social and material disadvantage at the small-area scale, all of which may “get under the skin” to influence ageing processes potentially across the life course.

There is a large and growing body of literature showing that the characteristics of where people reside matters for their health and the health deterioration process across adulthood. Evidence shows that, after accounting for individual-level socioeconomic characteristics, neighbourhood disadvantage was still associated with lower active life expectancy in the USA (Gill et al. 2021). People living in disadvantaged areas experience higher disability risk, worse self-rated health (SRH), multimorbidity, mortality, and more rapid health decline than residents of affluent areas (Ellaway et al. 2012; Prior et al. 2018; Peng et al. 2024; Rowley-Abel et al. 2025; Davies et al. 2025; Jivraj et al. 2025).

Recent large-scale studies have reported associations between higher neighbourhood deprivation and greater mid- and old-life cognitive impairment, neurodegeneration, cerebrovascular disease, and increased dementia risk (Hofbauer and Rodriguez 2021; Tan and Tan 2023; Baranyi et al. 2024; Low et al. 2025). Living in disadvantaged neighbourhoods is found to have a negative effect on total brain volume, grey matter, white matter integrity, hippocampal volume, and cognitive function, while such associations were weaker for individuals from higher social classes measured by individual-level sociodemographic characteristics (Tan and Tan 2023; Baranyi et al. 2024).

Alongside cognitive and subjective measured health outcomes, attention has turned towards the possibility of neighbourhood deprivation shaping biological ageing. The concept of biological embedding posits that chronic exposure to stressors and structural disadvantage can alter epigenetic regulation, accelerate multi-system dysregulation, and increase physiological wear and tear (Krieger 2001; Hertzman and Boyce 2010). DNA methylation (DNAm)-based ageing measures, such as DunedinPACE capture multi-system ageing processes and have shown strong predictive validity for morbidity, functional decline, and mortality (Duan et al. 2022; Kusters and Horvath 2024), which represent a promising tool for studying how neighbourhood conditions may influence biological ageing. Emerging studies have suggested that residents of deprived communities exhibit accelerated DNAm age (Reuben et al. 2020; Lawrence et al. 2020; Lei et al. 2022; Kamis et al. 2025). Because neighbourhood disadvantage has been shown to have distinct associations with health outcomes across different populations or subgroups (Chamberlain et al. 2022; Tan and Tan 2023; Baranyi et al. 2024), it is likely that associations between area-level deprivation and biological ageing also vary across subgroups. Whether DNAm-based ageing measures capture cumulative area-level exposure beyond individual socioeconomic position therefore remains insufficiently tested, and vulnerable subgroups remain unidentified.

Despite this growing literature, several gaps remain in exposure measurement, health outcome coverage, and study design, limiting understanding of how neighbourhood deprivation shapes ageing. First, studies often rely on a single area deprivation index. Area deprivation indices capture different dimensions of material and social disadvantage, so reliance on a single measure limits comparability and may underestimate contextual effects (Mogin et al. 2025). Examining multiple indices within the same population allows assessment of the robustness of contextual associations and provides insight into which dimensions of deprivation are most relevant for ageing outcomes. Second, most studies examine only one domain of ageing in isolation. Literature suggests that neighbourhood deprivation influences multiple dimensions of ageing, but the evidence remains fragmented across cognitive, subjective, and biological domains, so it is unclear whether area-level associations are domain-general or domain-specific. Relatively few studies have examined area deprivation using measures that assess “under-the-skin” biological ageing alongside cognitive and subjective health outcomes in the same population sample and rarely cover the wide age range of adults beyond those in the over-retirement-age group. Third, previous research has typically only adjusted for individual-level sociodemographic factors, and few additionally examine whether the association between area deprivation and biological ageing varies across population subgroups, which limits understanding of whether area-level influences operate uniformly or whether certain groups of people are more vulnerable to area-level disadvantage. Finally, most studies are cross-sectional, which limits insight into whether area deprivation is associated only with baseline health differences or also with divergent ageing trajectories over time.

In this paper, we address several of these gaps by examining the association between area deprivation and ageing measures in different domains, including molecular-level biological ageing, organ-system biological risk, cognitive performance, subjective physical functioning, and general health across the entire adult age span in the UK. By evaluating three deprivation indices separately, we test the robustness of contextual associations across distinct operationalisation of area-level socioeconomic conditions. Our analysis involves adjustments for individual-level sociodemographic factors, enabling a clearer assessment of whether area-level associations persist after accounting for measured compositional differences. We also elaborate these models to perform subgroup analysis to test whether area-level associations differ by compositional attributes of residents. Finally, by leveraging repeated measures of subjective functioning, we examine whether area deprivation is associated not only with baseline levels of health but also with the pace and shape of change in health trajectories over time.

## Methods

### Study design and participants

This study used data from the UK Household Longitudinal Study (UKHLS). The main UKHLS survey started in 2009 and has gathered data on an annual basis, with 15 waves completed to date. Approximately five months after waves 2 and 3 of the main survey, participants aged 16 years and older received a Health Assessment, during which trained nurses collected a range of biomedical measures and other health indicators. A total of 13,286 participants provided blood, and due to financial constraints, DNAm data were collected from a subsample of 3,654 participants, which was restricted to those of White/European ethnicity (Understanding Society 2025a, b). Respondents from Northern Ireland were not included in the Health Assessment; thus, our participants are those from Great Britain only. For the cross-sectional analyses, we used data from this Health Assessment (2010–2012). For the longitudinal analyses, we used data from waves 3 to 15 of the main survey, spanning 2012 to 2024.

Although there was some missingness in ageing measures collected during the Health Assessment (Table 1), because the overall missingness on area deprivation indices and covariates for data from Health Assessment was less than 1%, imputation is unnecessary and complete case analysis was conducted. After exclusion of individuals with missing data in any baseline covariates (n = 52) or area deprivation data (n = 5), the analytic sample comprised 13,174 participants from the Health Assessment, and 3,636 for DNAm-based measures. In the longitudinal analysis, sample sizes varied across ageing measures due to outcome-specific missingness, which was not imputed. There were 118,862 observation points available in the subsequent 13 waves (waves 3–15) for longitudinal analysis (see Supplementary Table A.1).

**Table 1:** Baseline characteristics of participants (2010–2012)

Outcomes	
DunedinPACE^a^, Mean (SD)	1.06 (0.14)
*Missing, N (%)*	*0 (0.00)*
BHS (0–16)^b^, Mean (SD)	3.63 (2.30)
*Missing, N (%)*	*3713 (28.18)*
Cognitive function (Memory domains)^b^, Mean (SD)	0.74 (0.14)
*Missing, N (%)*	*1016 (7.71)*
Cognitive function (Non-memory domains)^b^, Mean (SD)	0.56 (0.12)
*Missing, N (%)*	*1299 (9.86)*
PCS^b^, Mean (SD)	49.44 (11.12)
*Missing, N (%)*	*415 (3.15)*
SRH^b^, Mean (SD)	3.41 (1.09)
*Missing, N (%)*	*0 (0.00)*
Exposures	
Quintiles of IMD (England)^c^, N (%)	
1 (most deprived)	1805 (16.31)
2	1958 (17.69)
3	2365 (21.37)
4	2489 (22.49)
5 (least deprived)	2449 (22.13)
Quintiles of Townsend index, N (%)	
1 (most deprived)	1761 (13.37)
2	2377 (18.04)
3	2802 (21.27)
4	3148 (23.90)
5 (least deprived)	3086 (23.43)
Quintiles of Carstairs index, N (%)	
1 (most deprived)	1771 (13.44)
2	2471 (18.76)
3	2888 (21.92)
4	3032 (23.02)
5 (least deprived)	3012 (22.86)
Covariates	
Sex, N (%)	
Male	5866 (44.53)
Female	7308 (55.47)
Age groups, N (%)	
16–39	3369 (25.57)
40–52	3429 (26.03)
53–65	3293 (25.00)
66 +	3083 (23.40)
Highest qualification, N (%)	
Degree/professional or higher	4568 (34.67)
School level	6722 (51.03)
No qualification	1884 (14.30)
Occupation, N (%)	
Management and professional	3170 (24.06)
Intermediate	1788 (13.57)
Routine	2495 (18.94)
Retired	3738 (28.37)
Full-time student and others	1983 (15.05)
Quintiles of individual monthly disposable income, N (%)	
1 (highest)	2640 (20.04)
2	2639 (20.03)
3	2639 (20.03)
4	2631 (19.97)
5 (Lowest)	2625 (19.93)
Urbanity, N (%)	
Urban	9761 (74.09)
Rural	3413 (25.91)
Partnership status, N (%)	
Married or cohabitation	9013 (68.42)
Others	4161 (31.59)
Ethnicity, N (%)	
White	12572 (95.43)
Others	602 (4.57)

SD: standard deviation; N: sample size

^a^Epigenetic sample (*n* = 3636)

^b^Blood sample (*n* = 13,174)

^c^There were 11,066 participants in England, 950 in Wales, and 1158 in Scotland

### Measures

Area deprivation is a multidimensional evaluation of small geographical areas’ socioeconomic conditions. Small geographical areas were defined by the official spatial scale of lower-layer super output areas (LSOAs, and the equivalent Scottish Data Zones) using the 2011 Census boundaries. We assigned to each participant at Health Assessment the deprivation score of their residential small area, using three different indices: IMD, Townsend index, and Carstairs index. Because each country computes its own version of multiple deprivation index with different weights and different domain scoring, multiple deprivation indexes are not directly comparable across nations. The inclusion of the Townsend index and the Carstairs index makes the area deprivation comparable across Great Britain. The IMD is the official small-area measure of multiple deprivation in England, which combines seven domains (income; employment; education, skills and training; health and disability; crime; barriers to housing and services; living environment) (Ministry Of Housing, Communities & Local Government (2018 to 2021) 2015). Townsend index is based on the proportion of homeownership, unemployment, car ownership, and house overcrowding (UK Data Service 2022). Carstairs index is also formed from four census-derived measures, including the proportion of low social class, unemployed males, car ownership, and house overcrowding (Wheeler 2019; Brown et al. 2022). IMD offers a comprehensive composite of multiple deprivation domains, based on more factors included compared to the other two. Different indices capture overlapping but not identical constructs of area-level inequality. For example, the Townsend primarily captures material deprivation rather than broader social deprivation. Thus, using multiple indices reduce risk of measurement artefacts unique to a single deprivation metric, and increases confidence in contextual effects if associations are consistent across measures. We categorised each index into quintiles (from most deprived to least deprived), with quintile 5 indicating participants living in the most affluent areas.

We aim to assess the cognition, physical, and physiological manifestations of ageing processes, so ageing measures covered biological, cognitive, and subjective functioning domains. Apart from subjective functioning measures, all other ageing measures were collected at a single time point and were therefore used only for cross-sectional analyses. Ageing measures were selected and calculated following previously published research, which provides full details of the measurement protocols, variable selection, coding procedures, and score construction (Wang et al. 2026).

Biological ageing was measured using the DunedinPACE DNAm algorithm as molecular-level measures capture systemic rate of biological ageing, and the Biological Health Score (BHS) as organ-system-level measures reflects multi-system physiological dysregulation. DunedinPACE was used as a continuous measure of the pace of biological ageing (years of accelerated ageing per chronological year) (Belsky et al. 2022). All models for the DunedinPACE DNAm algorithm were additionally adjusted for technical covariates (barcode and various cell composition estimates) (Understanding Society 2025b). The composite organ-system biological ageing measure, BHS, indicates people’s physiological wear and tear. BHS was constructed from 16 biomarkers across six physiological systems, and as the sum across all ageing-related biomarkers of binary variables indicating if a person belongs to the “at-risk” quartiles of each biomarker (Wang et al. 2026). For biological ageing measures, a higher value means biologically older.

For cognitive function, we combined episodic and working memory indicator tests results into a “memory domain” score, and fluid reasoning, category fluency, and numeracy indicators into a “non-memory domain” score (Wang et al. 2026), considering different domains of cognitive function decline at different speeds across the life course (Salthouse 2009; Deary et al. 2009; Whitley et al. 2016). All indicators were min–max-normalised, and then, mean scores were computed for two domains separately.

Subjective functioning measures included physical health assessed using the physical component score (PCS) of the Short Form 12-item Survey (SF-12), and SRH which indicates self-reported general health status on a 5-point scale (1 = poor to 5 = excellent), and were collected at waves 2 and 3 for cross-sectional analyses and at subsequent waves 3–15 for longitudinal analyses. SRH was treated as a continuous variable in our primary analysis.

The higher scores for all cognitive function and subjective functioning measures indicate better functioning.

Covariates included sex; age group (participants’ age tertile as cut-off point, and categorised participants to 16–39, 40–52, 53–65, and 66 +); quintiles of individual monthly disposable income; occupation (based on the 3 class National Statistics Socio-economic classification (NS-SEC): “management & professional”, “intermediate”, “routine”; plus “retired”, “full-time student and others”); education is classified as highest qualification (“degree/professional or higher” (higher than A-levels or equivalent), “school level” (up to A-levels or equivalent), and “no qualification”); urbanity; partnership status; and ethnicity. In sensitivity analyses, health behaviours and long-standing illness/disability (long-standing physical or mental impairment, illness, or disability over a period of at least 12 months) were added to reduce potential confounding by health selection. Health behaviours included smoking status (“never smoker”, “ex-smoker”, and “current smoker”), drinking frequency of last week (“not in past week”, “1–2 days”, “3–4 days”, “5 + days”), physical activity (“doing no sport at all”, “mild”, “moderate”, and “very active”). All those covariates measured at baseline (2010–2012).

### Statistical analyses

We estimated cross-sectional associations using linear regression models with standard errors clustered at the LSOA level. To explore shared contextual exposure that occurs at area scale, LSOAs were chosen as the unit of clustering rather than household clustering.

Sensitivity analyses for cross-sectional analysis included (1) models without geographic clustering; (2) clustering at a larger geographic scale (middle-layer super output areas (MSOAs), formed by grouping together LSOAs, typically cover on average from 5000 to 15,000 people (2000–6000 households) compared with 1000–3000 people (400–1200 households) for LSOAs) to avoid unstable cluster-level variance estimation due to some LSOAs with very few participants (less than 2), especially for DNAm subsample; (3) use multilevel random intercepts linear regression models with LSOA at level 2 to account for geographical clustering rather than cluster-robust standard errors; (4) additional adjustment for long-standing illness/disability and health behaviours. Because health behaviours and long-standing illness may plausibly function either as baseline confounders through health selection or as downstream mediators of neighbourhood deprivation, we treated them as sensitivity covariates rather than primary adjustment variables; (5) test the association using available PCS and SRH data from waves 4 (2012–2014), 5 (2013–2015), 6 (2014–2016), and 7 (2015–2017) of the UKHLS main survey separately to test for potential reverse causation in the primary analysis; (6) although treating SRH as a continuous variable has demonstrated good construct validity in prior research (Cullati et al. 2020), we additionally re-analysed SRH as an ordinal outcome in sensitivity analyses to assess the robustness of results to alternative outcome specification.

Subgroup analysis for cross-sectional analysis was carried out to investigate whether area-level associations differ by compositional characteristics. We conducted subgroup analyses by sex, age groups, education, occupation, income quintiles, urbanity, partnership status, and ethnicity. Wald tests were used to evaluate the statistical significance of the interaction effects, and moderation analyses were used to explore potential subgroup differences.

For longitudinal analyses, we estimated associations between baseline area deprivation and trajectories of subjective functioning using growth curve models specified as multilevel linear models with standard errors clustered at the LSOA level. Repeated observations over time were nested within individuals, with each wave observation treated as level 1 and participants as level 2. Random intercepts and random slopes for data collection wave were included to account for between-individual heterogeneity in baseline levels and rates of change, and an unstructured variance–covariance matrix was specified for the random effects to allow for flexible correlation between intercepts and slopes. Time was modelled as years since baseline (wave 3), and all longitudinal models were adjusted for baseline covariates. The final models additionally included a quadratic time term (wave squared) and interaction terms between wave and area deprivation indices to assess nonlinear trajectories and differential rates of change by area deprivation.

All analyses were conducted using Stata (version 19.0). To account for multiple comparisons, Bonferroni correction was applied, and Bonferroni-adjusted significance threshold was defined as 0.05 divided by the number of deprivation indices tested within each outcome domain.

## Results

### Participant baseline characteristics

Among the 13,174 participants in the Health Assessment, their mean age was 51.63 years (SD 17.14), and 55.47% were female. The distribution of quintiles of deprivation indices from most deprived to least deprived were 16.31%, 17.69%, 21.37%, 22.49%, and 22.13% of participants for IMD; 13.37%, 18.04%, 21.27%, 23.90%, and 23.43% of participants for Townsend index; and 13.44%, 18.76%, 21.92%, 23.02%, 22.86% of participants for Carstairs index (Table 1). In Table A.2 (see Supplementary tables file), we can see that for Health Assessment, the correlations between quintiles of IMD, Townsend index, and Carstairs index were strong (*r* value range: 0.830–0.906). This indicates that while the indices capture closely related dimensions of area deprivation, they are not interchangeable. For the DNAm subsample (n = 3636), analytic samples were smaller but showed similar sociodemographic patterns (see Supplementary Table A.3).

**Table 2:** Cross-sectional associations between area deprivation indices and biological ageing measures, clustering of individuals within LSOAs

	DunedinPACE		BHS	
	Coef. (95% CI)	*P* value	Coef. (95% CI)	*P* value
Quintiles of IMD (England)				
1 (most deprived)	Reference		Reference	
2	− 0.034 (− 0.050 to − 0.019)	** < 0.001**	− 0.231 (− 0.419 to − 0.042)	0.016
3	− 0.029 (− 0.044 to − 0.013)	** < 0.001**	− 0.359 (− 0.538 to − 0.180)	** < 0.001**
4	− 0.046 (− 0.061 to − 0.030)	** < 0.001**	− 0.435 (− 0.616 to − 0.253)	** < 0.001**
5 (least deprived)	− 0.045 (− 0.061 to − 0.030)	** < 0.001**	− 0.603 (− 0.785 to − 0.422)	** < 0.001**
Observations (n)	2903		8,018	
Quintiles of Townsend index				
1 (most deprived)	Reference		Reference	
2	− 0.016 (− 0.032 to − 0.001)	0.036	− 0.119 (− 0.299 to 0.060)	0.193
3	− 0.023 (− 0.039 to − 0.008)	**0.002**	− 0.360 (− 0.533 to − 0.186)	** < 0.001**
4	− 0.036 (− 0.051 to − 0.020)	** < 0.001**	− 0.374 (− 0.550 to − 0.199)	** < 0.001**
5 (least deprived)	− 0.047 (− 0.062 to − 0.031)	** < 0.001**	− 0.531 (− 0.709 to − 0.353)	** < 0.001**
Observations (n)	3636		9461	
Quintiles of Carstairs index				
1 (most deprived)	Reference		Reference	
2	− 0.020 (− 0.035 to − 0.004)	0.011	− 0.239 (− 0.418 to − 0.059)	0.009
3	− 0.027 (− 0.042 to − 0.012)	** < 0.001**	− 0.390 (− 0.569 to − 0.211)	** < 0.001**
4	− 0.038 (− 0.053 to − 0.023)	** < 0.001**	− 0.449 (− 0.625 to − 0.272)	** < 0.001**
5 (least deprived)	− 0.059 (− 0.074 to − 0.043)	** < 0.001**	− 0.667 (− 0.852 to − 0.482)	** < 0.001**
Observations (n)	3636		9461	

Two-sided *P* value less than 0.05 would be considered statistically significant, and statistically significant *P* value after Bonferroni correction was marked as bold. Covariates’ coefficients are not shown for the sake of brevity

Coef.: coefficient; CI: confidence interval

Adjusted for technical covariates (for DunedinPACE DNAm algorithm only), ethnicity (not for DunedinPACE DNAm algorithm), age groups, sex, education, occupation, income quintiles, urbanity, partnership status

### Cross-sectional associations between area deprivation indices and ageing measures

Area deprivation measured by IMD, Townsend, and Carstairs indexes was associated with biological ageing, cognitive function, and subjective functioning measures. Across all three area deprivation indices, people living in more affluent areas had consistently slower biological ageing and better cognitive performance and subjective functioning, with strong and consistent social gradients. Such graded trends persisted across all quantiles for all ageing domains, supporting a graded, monotonic association consistent with a dose–response pattern.

For molecular- and organ-system-level ageing measures, greater area deprivation was associated with accelerated biological ageing measured by DunedinPACE and BHS separately (Table 2). For example, participants living in the least deprived areas (IMD quintile 5) had significantly lower DunedinPACE values (coefficient = − 0.045, 95% CI = − 0.061 to − 0.030, *p* < 0.001) compared with those in the most deprived areas. Patterns were similar when deprivation was measured using the Townsend index (coefficient = − 0.047, 95% CI = − 0.062 to − 0.031, *p* < 0.001) and Carstairs index (coefficient = − 0.059, 95% CI = − 0.074 to − 0.043, *p* < 0.001). The pattern was graded, with consistent reductions in DunedinPACE values from quintile 2 through quintile 5 of the Townsend and Carstairs indexes. Similarly, BHS showed higher biological risk scores in more deprived areas and had a graded pattern with consistent reductions in BHS values from more deprived to more affluent quintiles for all area deprivation indices.

Living in more deprived areas was associated with worse cognitive functioning (Table 3). For example, living in the most affluent areas was associated with better cognitive performance in the memory domain (coefficient = 0.039, 95% CI = 0.029 to 0.048, *p* < 0.001) and non-memory domain (coefficient = 0.036, 95% CI = 0.029 to 0.044, *p* < 0.001) compared with those in the most deprived quintile measured by IMD. Associations of similar magnitude were observed for area deprivation measured by Townsend and Carstairs indexes. In addition, cognitive function demonstrated clear gradients across both memory and non-memory domains.

**Table 3:** Cross-sectional associations between area deprivation indices and cognitive function, clustering of individuals within LSOAs

	Cognitive function (memory domains)		Cognitive function (non-memory domains)	
	Coef. (95% CI)	*P* value	Coef. (95% CI)	*P* value
Quintiles of IMD (England)				
1 (most deprived)	Reference		Reference	
2	0.021 (0.011 to 0.030)	** < 0.001**	0.013 (0.006 to 0.021)	** < 0.001**
3	0.034 (0.024 to 0.044)	** < 0.001**	0.026 (0.019 to 0.034)	** < 0.001**
4	0.032 (0.022 to 0.042)	** < 0.001**	0.027 (0.019 to 0.034)	** < 0.001**
5 (least deprived)	0.039 (0.029 to 0.048)	** < 0.001**	0.036 (0.029 to 0.044)	** < 0.001**
Observations (n)	10,183		9,952	
Quintiles of Townsend index				
1 (most deprived)	Reference		Reference	
2	0.020 (0.010 to 0.029)	** < 0.001**	0.015 (0.007 to 0.022)	** < 0.001**
3	0.024 (0.015 to 0.034)	** < 0.001**	0.020 (0.013 to 0.028)	** < 0.001**
4	0.032 (0.023 to 0.042)	** < 0.001**	0.029 (0.021 to 0.036)	** < 0.001**
5 (least deprived)	0.038 (0.029 to 0.048)	** < 0.001**	0.035 (0.027 to 0.042)	** < 0.001**
Observations (n)	12,158		11,875	
Quintiles of Carstairs index				
1 (most deprived)	Reference		Reference	
2	0.019 (0.009 to 0.028)	** < 0.001**	0.007 (-0.000 to 0.015)	0.056
3	0.025 (0.016 to 0.035)	** < 0.001**	0.019 (0.012 to 0.026)	** < 0.001**
4	0.033 (0.024 to 0.043)	** < 0.001**	0.027 (0.019 to 0.034)	** < 0.001**
5 (least deprived)	0.044 (0.035 to 0.054)	** < 0.001**	0.039 (0.032 to 0.047)	** < 0.001**
Observations (n)	12,158		11,875	

Two-sided *P* value less than 0.05 would be considered statistically significant, and statistically significant *P* value after Bonferroni correction was marked as bold. Covariates’ coefficients are not shown for the sake of brevity

Coef.: coefficient; CI: confidence interval

Adjusted for ethnicity, age groups, sex, education, occupation, income quintiles, urbanity, partnership status

Subjective functioning measured by PCS and SRH also exhibited deprivation gradients, and both PCS and SRH increased across deprivation quintiles (Table 4). Irrespective of the deprivation metric used, residents from deprived areas reported worse self-perceived physical health and general health compared with their counterparts from less deprived areas. For instance, relative to the most deprived IMD quintile, PCS was 3.536 higher (95% CI = 2.849 to 4.222, *p* < 0.001) in the least deprived quintile. Similarly, the associations were observed for Carstairs (coefficient = 3.315, 95% CI = 2.618 to 4.011, *p* < 0.001) and Townsend (coefficient = 2.716, 95% CI = 2.031 to 3.402, *p* < 0.001).

**Table 4:** Cross-sectional associations between area deprivation indices and subjective functioning measures, clustering of individuals within LSOAs

	PCS		SRH	
	Coef. (95% CI)	*P* value	Coef. (95% CI)	*P* value
Quintiles of IMD (England)				
1 (Most deprived)	Reference		Reference	
2	1.636 (0.920 to 2.353)	** < 0.001**	0.198 (0.130 to 0.267)	** < 0.001**
3	2.005 (1.290 to 2.721)	** < 0.001**	0.229 (0.161 to 0.298)	** < 0.001**
4	2.369 (1.679 to 3.059)	** < 0.001**	0.279 (0.212 to 0.346)	** < 0.001**
5 (Least deprived)	3.536 (2.849 to 4.222)	** < 0.001**	0.352 (0.283 to 0.421)	** < 0.001**
Observations (n)	10,708		11,066	
Quintiles of Townsend index				
1 (Most deprived)	Reference		Reference	
2	0.880 (0.182 to 1.579)	0.014	0.160 (0.093 to 0.227)	** < 0.001**
3	1.570 (0.900 to 2.239)	** < 0.001**	0.196 (0.130 to 0.261)	** < 0.001**
4	1.894 (1.210 to 2.577)	** < 0.001**	0.268 (0.201 to 0.334)	** < 0.001**
5 (Least deprived)	2.716 (2.031 to 3.402)	** < 0.001**	0.328 (0.260 to 0.395)	** < 0.001**
Observations (n)	12,759		13,174	
Quintiles of Carstairs index				
1 (Most deprived)	Reference		Reference	
2	0.945 (0.247 to 1.642)	0.008	0.173 (0.106 to 0.239)	** < 0.001**
3	2.119 (1.440 to 2.798)	** < 0.001**	0.250 (0.184 to 0.316)	** < 0.001**
4	2.245 (1.565 to 2.925)	** < 0.001**	0.272 (0.205 to 0.338)	** < 0.001**
5 (Least deprived)	3.315 (2.618 to 4.011)	** < 0.001**	0.387 (0.318 to 0.457)	** < 0.001**
Observations (n)	12,759		13,174	

Two-sided *P* value less than 0.05 would be considered statistically significant, and statistically significant *P* value after Bonferroni correction was marked as bold. Covariates’ coefficients are not shown for the sake of brevity

Coef.: coefficient; CI: confidence interval

Adjusted for ethnicity, age groups, sex, education, occupation, income quintiles, urbanity, partnership status

Our results were robust to multiple sensitivity checks (see Supplementary Table B.1 to Table B.6), which yielded similar associations and clear social gradient patterns.

### Subgroup analyses for cross-sectional associations

Stratified analyses revealed broadly consistent patterns: the association between area deprivation and worse ageing outcomes persisted across most subgroups (see Supplementary Table C.1 to Table C.18). Most interaction p values were greater than 0.05, especially for the association between IMD with biological ageing measures and cognitive functioning, indicating that the deprivation gradient was broadly similar across subgroups. No strong effect modification was observed for molecular- and organ-system-level ageing measures (see Supplementary Table C.1 to Table C.6), except a somewhat weak association observed for DunedinPACE in the youngest (16–39 years) and oldest (66 + years) age groups. Effect modification by education and occupation that survived Bonferroni correction was observed for the association between area deprivation, measured by Townsend and Carstairs indexes, but not for IMD, and cognitive function in memory and non-memory domains (see Supplementary Table C.7 to Table C.12). Some age variation was detected for subjective functioning measures, and education and occupation were observed as effect modification (see Supplementary Table C.13 to Table C.18).

### Longitudinal associations between area deprivation indices and subjective functioning

**Fig. 1:**
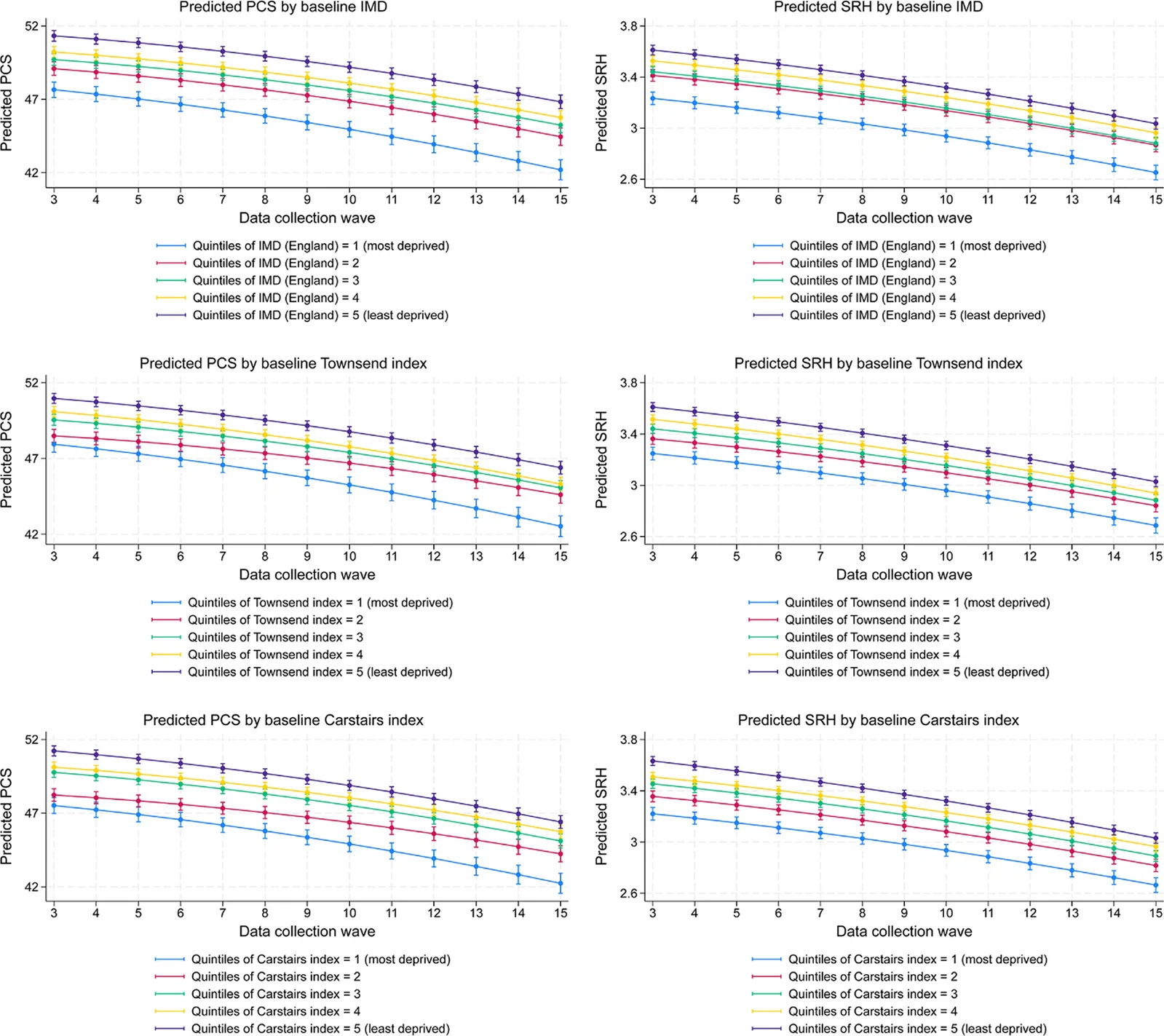
Predicted trajectories of self-reported health functioning

Longitudinal analyses showed that baseline area deprivation was associated with both lower initial levels and, in some instances, differential trajectories of subjective functioning over time (Fig. 1 and Supplementary Table D.1). For PCS, significant deprivation-by-time interactions were observed for Townsend and Carstairs indices, indicating widening inequalities over follow-up, whereby participants residing in more affluent areas experienced slower declines in physical functioning. In contrast, IMD was associated primarily with persistent baseline inequalities rather than significant divergence over time. For SRH, deprivation gradients remained relatively stable over follow-up, indicating persistent inequalities rather than substantial widening.

## Discussion

We found that area deprivation was consistently associated with independent measures of biological ageing, cognitive performance, and subjective health and functioning. This relationship appeared across multiple independently measured health domains, using three different deprivation indices, and displayed highly consistent cross-sectional social gradients. The baseline associations were graded across deprivation quintiles, demonstrating a monotonic pattern of poorer health performance with increasing area-level disadvantage. However, our longitudinal findings were less consistent such that area deprivation does not universally widen health inequalities over time. Longitudinal impacts are outcome- and index-specific: baseline area deprivation is associated with widening, divergent trajectories of physical functioning when captured by indices focusing primarily on material and occupational assets (Townsend and Carstairs indices), but is characterised by stable, persistent inequalities over time when examining self-rated general health.

Different ageing markers represent complementary layers of ageing: DunedinPACE captures molecular ageing velocity, BHS reflects multi-system dysregulation, cognitive function represents neurocognitive ageing, PCS reflects physical functional health, and SRH is a global perception of health status. Convergence of cross-sectional effects across these domains suggests that deprivation is embedded broadly across multiple domains of ageing across the entire adult age span. By adjusting for a broad set of individual-level factors, we assessed whether associations persist after accounting for measured compositional factors in residents’ socioeconomic and demographic characteristics. Our findings align with prior research documenting neighbourhood effects on health and cognition for older people (Hofbauer and Rodriguez 2021; Tan and Tan 2023; Low et al. 2025). Our results extend previous findings in wider age groups of the UK population by demonstrating that area deprivation is also associated with biological ageing measures across the entire adult age span, consistent with emerging evidence that social and environmental disadvantages correlate with blood and epigenetic markers of ageing (Lawrence et al. 2020; Lei et al. 2022; Holman et al. 2022; Kamis et al. 2025).

Our longitudinal analyses extend the cross-sectional evidence by demonstrating that area deprivation is not only associated with worse baseline levels of subjective functioning but also with divergent health trajectories over time. Participants residing in more deprived areas experienced persistently lower PCS and SRH scores across subsequent waves. This pattern is consistent with prior findings showing that neighbourhood disadvantage may influence not only baseline health differences but also the pace of ageing and health decline over time. For example, analyses of the 1958 National Child Development Study reported that greater deprivation in adolescence was associated with worsening self-rated health across adulthood, with cumulative exposure explaining changes beyond concurrent measures (Jivraj et al. 2021). Research in the Lothian Birth Cohort shows that mid-to-late adulthood deprivation predicts lower cognitive function at age 70 and faster cognitive decline over 12 years (Baranyi et al. 2023). Evidence from the English Longitudinal Study of Ageing also reveals that living in more deprived areas increases the risk of frailty over extended follow-up (Maharani et al. 2023). Our findings complement and extend the literature by focusing explicitly on subjective functioning trajectories across a wider adult age range and by testing multiple deprivation indices. We find the area-deprivation gradient widened over time for the PCS, whereas it remained parallel and stable for SRH. PCS serves as a direct indicator of somatic functional limitations, physical bodily capacities, and mobility constraints, whereas SRH is a broader subjective assessment that incorporates physical health, mental well-being, social comparisons, expectations, and adaptation processes (Kraft and Kraft 2021). As individuals age within socio-spatially segregated environments, they may adjust their health expectations to align with local community norms and peer references. Individuals may adapt their overall health perceptions despite deteriorating physical functioning, which could attenuate widening inequalities in SRH over time.

Residential inequalities across small geographic areas can act as structural mechanisms that produce differential exposures to physical and/or social environments (Macintyre et al. 2002), thus affecting ageing in a graded way. The cross-sectional and longitudinal patterns suggest a cumulative and potentially compounding influence of contextual disadvantage. Our findings reveal that area-level influences on ageing operate not only at a single time point but also in the persistence or widening of health inequalities across follow-up. The mechanisms through which neighbourhood deprivation may affect ageing are likely multifactorial and cumulative. Environmental hazards, limited access to healthy food and safe spaces for physical activity, reduced social cohesion, inadequate services, etc., all concentrated in more deprived areas, may increase biological wear and tear and cumulative physiological dysregulation. For example, a study conducted on a UK population found that the association between area deprivation and health outcomes can be mediated by physical living condition characteristics (including air pollution, exposure to green space, and proximity to waste and industrial facilities) (Chaparro et al. 2018). Exposure to contextual disadvantage was found to trigger chronic stress accumulation across the life course, resulting in dysregulation of multiple bodily systems, which may increase the ageing process (Baranyi et al. 2024; Kamis et al. 2025).

However, our findings should not be interpreted as evidence that neighbourhood deprivation itself causes accelerated ageing. Residential environments reflect both contextual exposures and selective residential mobility, and individuals may not randomly distribute across neighbourhoods. Consequently, observed associations likely reflect both place-based influences and compositional processes. The sensitivity analyses provide critical insights into the intersection of context and composition. When our cross-sectional models were adjusted for baseline health behaviours and long-standing physical or mental illness, the area-deprivation coefficients were noticeably attenuated, though most gradients maintained statistical significance. This pattern indicates that individual composition and pre-existing health status account for a meaningful portion of the area-level variations. In accordance with theories of health selection and selective sorting, individuals experiencing chronic illnesses or possessing behavioural risk profiles often face structural constraints that restrict residential choice, leading to their concentration in highly deprived locales (Darlington-Pollock and Norman 2022). Therefore, future research is needed, and our models do not completely isolate a pure contextual space independent of the people living there; instead, they reflect a complex configuration where structural area-level disadvantages and individual compositional sorting processes reinforce one another.

Subgroup results suggest that area-level associations operate broadly across population groups, with few exceptions for people in different age groups, education or occupation backgrounds. Additional stratification by individual-level sociodemographic factors did not materially change the direction or size of most associations, suggesting that area-level deprivation exerts broadly shared influences on ageing that are not confined to narrowly defined social subgroups. However, the absence of strong moderation does not imply uniform vulnerability. Individual-level factors may not act independently but intersect to produce distinct patterns of exposure and resilience, drawing on intersectionality theory (Bambra 2022; Holman et al. 2022). The intersection of individual-level disadvantage, rather than single factors, may amplify the influence of area deprivation on ageing; thus, here testing single factors separately may yield null findings. In addition, our stratified models tested factors one at a time, which may overlook compounded disadvantage among individuals who experience multiple forms of marginalisation. Future work should test interactions between area deprivation and individual socioeconomic position using suitable intersectional analytic frameworks.

However, although the subgroup difference by individual socioeconomic position was not found for “under-the-skin” ageing measures, the education and occupation moderation effects were observed for cognition and subjective functioning. Viewed through the lens of the social model of disability theory, functional and subjective health outcomes may not be driven solely by internal somatic pathology; rather, they emerge from the structural interface between an individual’s personal resource reserves and the structural barriers present in their immediate residential space. Our findings suggest that individual-level socioeconomic assets may act as a buffer against neighbourhood adversity. Subjective health likely reflects not only internal bodily signals but also social comparison processes shaped by local norms, perceived resources, and expectations (Kraft and Kraft 2021). These socially embedded perceptions may be more responsive to educational or occupational differences and to the social character of neighbourhoods than molecular ageing biomarkers, which reflect underlying physiological processes.

Our findings underscore the importance of neighbourhood-level disadvantage as a structural determinant of ageing. Policies aimed at reducing area deprivation, such as improving housing quality, environmental conditions, local infrastructure, social cohesion, and removing structural barriers, may have downstream benefits by slowing biological ageing processes and altering adverse health trajectories, thereby attenuating inequalities in cognitive, functional, and overall health across adulthood. Moreover, because area deprivation affects multiple ageing domains, neighbourhood improvements may have spillover benefits and generate broad health returns, which represent an efficient strategy for minimising health inequalities across the life course.

This study has several limitations. A relatively small number of participants aged 80 and over at baseline restrict our ability to draw firm conclusions regarding extreme old age. The DNAm subsample was restricted to White/European participants, so we are unable to evaluate racial/ethnic heterogeneity in molecular-level biological ageing associations. Selective retention likely resulted in a healthier and socioeconomically advantaged analytic sample over follow-up, potentially leading to conservative estimates of deprivation gradients. Those may underestimate inequalities present in the wider population. In addition, cross-sectional data analyses for most ageing outcomes limit causal inference. The availability of repeated measures for subjective health outcomes strengthens inference by allowing examination of within-person change over time, partially mitigating concerns about reverse causation inherent in purely cross-sectional designs. Although we did later-wave analyses and longitudinal analyses for PCS and SRH to avoid potential reverse causation, this study remains observational. Further, this study relied on complete case analysis, which may introduce bias if missingness is related to unobserved characteristics associated with both area deprivation and ageing outcomes. However, missingness in area deprivation indices and baseline covariates was minimal, and outcomes were not imputed. Under standard missing-at-random assumptions, complete case analysis is unlikely to bias estimates of associations (Austin et al. 2021), although reduced precision remains possible. Residual confounding by unmeasured individual or family-level factors and by life-course residential histories is possible. Although we have checked that 90% of participants did not move home in at least 3 years, our study cannot address residential selection or reverse causation. Future research incorporating cumulative or historical exposure to deprivation will strengthen the inference. Our observational framework cannot fully account for selective residential mobility and genetic confounding. Shared genetic and early-life factors may influence both neighbourhood selection and ageing outcomes (Cronqvist et al. 2014; Finkel et al. 2025), so residual confounding cannot be excluded. Accordingly, our findings should be interpreted as associations rather than causal effects of neighbourhood deprivation. The deprivation indices we used in this study capture structural disadvantage at a single time point based on census data and do not reflect lifetime exposure or neighbourhood trajectories. The limitations in the granularity of deprivation indices may bias effect estimates. Although the use of multiple deprivation indices is a strength, these indices capture aggregated characteristics and cannot identify the specific neighbourhood features (for example, air quality, green space, neighbour cohesion) responsible for the observed associations. Possible circularity may exist when using IMD (which includes a health and disability domain) in models predicting health-related outcomes, but the inclusion of the Townsend and Carstairs indexes increases the reliability of the findings.

## Supplementary Information

Below is the link to the electronic supplementary material.

Supplementary file1 (XLSX 135 KB)

## Data Availability

The data that support the findings of this study are available from the UK Household Longitudinal Study (Understanding Society) via the UK Data Service (https://www.ukdataservice.ac.uk/). The datasets analysed during the current study include “Understanding Society: Waves 2-3 Nurse Health Assessment, 2010-2012” (https://datacatalogue.ukdataservice.ac.uk/studies/study/7251), “Understanding Society: Waves 1-15, 2009-2024: Special Licence Access, Census 2011 Lower Layer Super Output Areas” (https://datacatalogue.ukdataservice.ac.uk/studies/study/7248), “Understanding Society: Waves 1-15, 2009-2024: Special Licence Access, Census 2011 Middle Layer Super Output Areas” (https://datacatalogue.ukdataservice.ac.uk/studies/study/7249), and “Understanding Society: Waves 1-15, 2009-2024 and Harmonised BHPS: Waves 1-18, 1991-2009” (https://datacatalogue.ukdataservice.ac.uk/studies/study/6614). Access to the data is subject to registration and acceptance of the UK Data Service End User Licence Agreement.
